# On the relationship between research parasites and fairness in machine learning: challenges and opportunities

**DOI:** 10.1093/gigascience/giab086

**Published:** 2021-12-20

**Authors:** Nicolás Nieto, Agostina Larrazabal, Victoria Peterson, Diego H Milone, Enzo Ferrante

**Affiliations:** Research Institute for Signals, Systems and Computational Intelligence, sinc(i), FICH-UNL/CONICET, Santa Fe (3000), Argentina; Research Institute for Signals, Systems and Computational Intelligence, sinc(i), FICH-UNL/CONICET, Santa Fe (3000), Argentina; Instituto de Matemática Aplicada del Litoral, IMAL-UNL/CONICET, Santa Fe (3000), Argentina; Research Institute for Signals, Systems and Computational Intelligence, sinc(i), FICH-UNL/CONICET, Santa Fe (3000), Argentina; Research Institute for Signals, Systems and Computational Intelligence, sinc(i), FICH-UNL/CONICET, Santa Fe (3000), Argentina

**Keywords:** fairness, deep learning, machine learning

## Abstract

Machine learning systems influence our daily lives in many different ways. Hence, it is crucial to ensure that the decisions and recommendations made by these systems are fair, equitable, and free of unintended biases. Over the past few years, the field of fairness in machine learning has grown rapidly, investigating how, when, and why these models capture, and even potentiate, biases that are deeply rooted not only in the training data but also in our society. In this Commentary, we discuss challenges and opportunities for rigorous posterior analyses of publicly available data to build fair and equitable machine learning systems, focusing on the importance of training data, model construction, and diversity in the team of developers. The thoughts presented here have grown out of the work we did, which resulted in our winning the annual Research Parasite Award that *GigaScience*sponsors.

## Introduction

Machine learning (ML) algorithms make or support decisions that have strong implications for the lives of individuals and the community as a whole. ML-based systems drive autonomous vehicles, control weapons such as drones, diagnose medical conditions, make employment decisions, grant loans, and even help political candidates to win elections. These systems also have the capability of modifying our behaviour, influencing what we watch and buy, where we move, and even whom we date. Hence, it is of great importance that the decisions and recommendations made by these algorithms be fair, equitable, and free of biases that may favour certain subpopulations over others.

Recently, the research community of fairness in ML has shown that, contrary to popular belief about computer systems, these models can be far from objective and the decisions that they make can be strongly influenced—even unintentionally—by population demographic factors such as sex, ethnicity, or age, resulting in poor performance for specific subgroups [1,2]. The causes behind this phenomenon are multiple, and range from lack of diversity in the team of developers [3] to the technical design choices in terms of model architecture, objective functions, and training algorithms [4]. Another fundamental aspect is the data used to train these models. Because ML algorithms learn to find patterns and associations from what is called “training data”, their performance highly depends on how representative the subsample used to train the model is for the target population.

**Figure 1:**
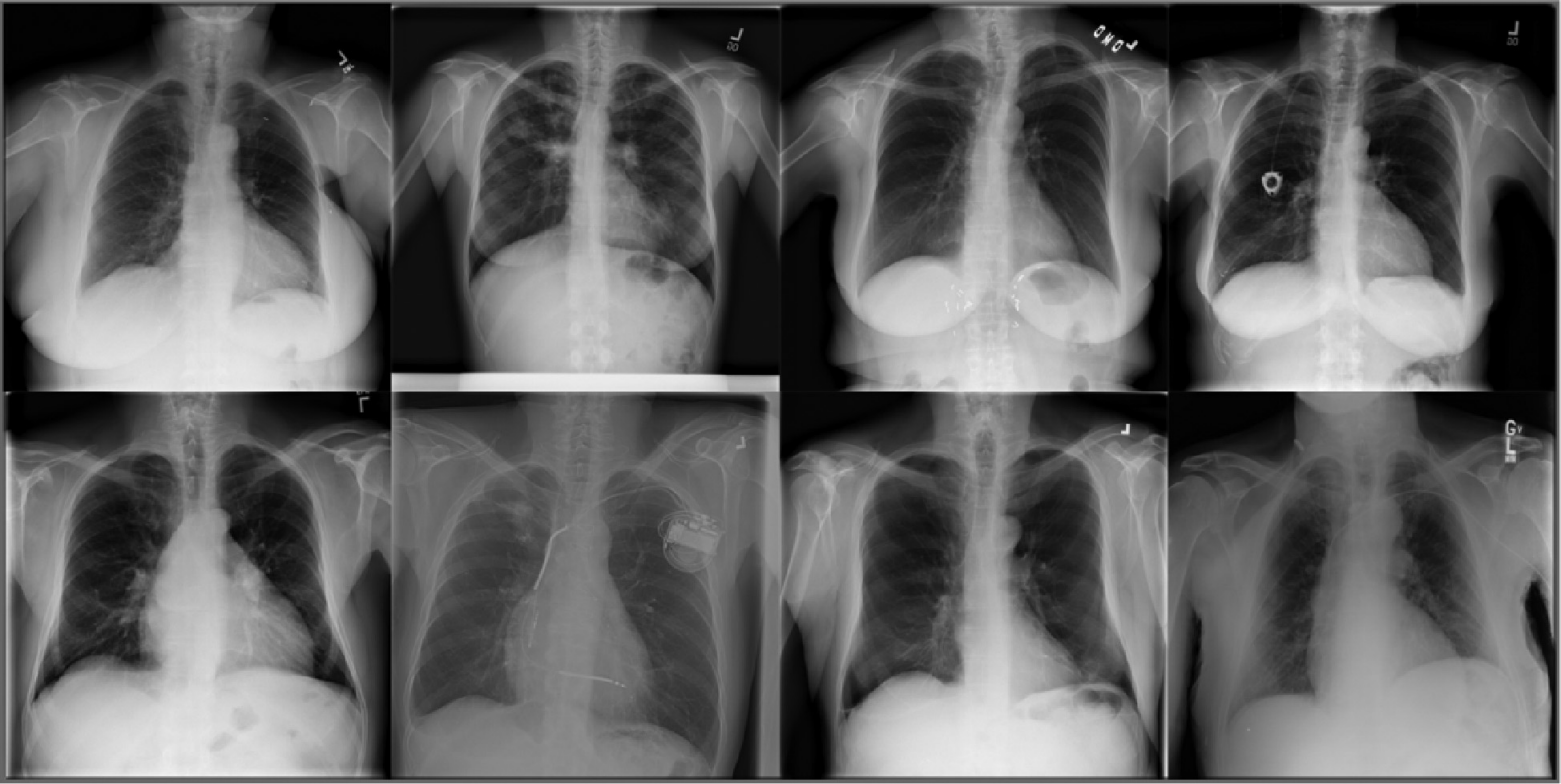
Some example of images from the public databases used in the awarded article [5].

It is well known that collecting and curating databases can be in itself a highly expensive task. As researchers coming from Latin America, we want to highlight the value of well-structured and documented publicly available datasets as an opportunity to carry out research that, many times, would not be possible otherwise for us (Fig. 1). However, even though we acknowledge the value of such datasets for democratizing research opportunities, we are also aware that they tend to reflect the reality of those places where they were acquired. As such, we envision the creation of local datasets as an occasion to truly democratize research and ensure, at the same time, the fairness of ML models for our local population.

Given all of this, our recent article [5]—for which we received the 2020 Junior Research Parasite Award and which is the *raison d’être* of this Invited Commentary—raises the alarm about potential biases that may emerge in the context of computer-aided diagnosis when training databases are not carefully designed and curated. Understanding the importance of the secondary uses of publicly available data is vital for eliminating the perpetuation of biases that can have a major negative impact on a variety of groups; thus, we are writing this commentary with the spirit of highlighting the challenges and opportunities of parasite studies in relation to building fair and equitable ML systems.

## Challenges and Opportunities for Research Parasites in the Building of Fair ML Systems

Secondary data analysis is generally related to the idea of using existing data collected by others to test new and different hypotheses. But in the context of fairness studies in ML, new opportunities emerge where secondary data analysis can offer even more than that. We can use existing datasets to (i) audit our models to ensure equitable results for minority subpopulations, (ii) assess (and improve) the performance of predictive systems under dataset shifts in a variety of application domains, (iii) generate counterfactual scenarios considering different intersectional axes of analysis, (iv) understand model behaviour under different deployment conditions, (v) and even perform data exploratory studies to discover potential biases in the sampling procedures. However, for this to be possible, the quality of such datasets needs to be guaranteed. Not only the data themselves but also the metadata have to be well curated and documented for these databases to be useful in subsequent studies [6].

The consequences of biased ML systems are easy to see especially in the case of human data. In such a scenario, if we want to audit a given system to ensure equitable results in terms of gender, ethnicity, or age, we need to have access to demographic attributes at the individual level. Although the utility of well-curated databases with disaggregated metadata for secondary analysis is clear, the release of individual information must comply with individual privacy policies. This trade-off between privacy and utility in data publishing [7] becomes especially important when constructing databases that incorporate quasi-identifiers (e.g., attributes like zip or postal codes, age, or sex) whose values, when combined, can potentially identify an individual. Anonymization techniques, which transform data samples to improve privacy, can be considered as potential solutions. However, as discussed in [7], because anonymization makes data more imprecise, it also causes losses in utility when compared with the case of publishing the non-anonymized entries. This trade-off must be considered to ensure ethical treatment of human data.

Another issue that appears when incorporating demographic attributes to the databases is related to the categories that need to be defined. There is no doubt about the value that should be assigned to age, for example; but this is not always the case for other demographic features. Different characteristics such as sexual orientation or gender identity tend to be fluid and sometimes difficult to “label” or quantify. In fact, these are prototypical instances of unobserved characteristics that are frequently missing in databases, either because they are unknown or, in some cases, because they are intrinsically unmeasurable [8]. These issues in measurability yield discrepancies and tension in how fairness is applied across different contexts ranging from credit scoring to healthcare [9] and spark interesting debates on how to address demographic disparities when we cannot see or measure these sensitive attributes. We believe this is an open question that can benefit from research parasite studies.

In line with these observations, Gebru et al. [10] propose the use of datasheets for datasets. They suggest that every dataset should be accompanied by a datasheet that documents its motivation, composition, collection process, recommended uses, and other important aspects, with the ultimate goal of increasing transparency and accountability within the community, mitigating unwanted biases in ML systems, and encouraging reproducibility of ML experiments. Datasheets for datasets constitute a useful tool that can increase the value of published databases and help the community of research parasites to perform rigorous secondary data analyses.

## It Is Not All About Data

Although it is true that data play an important role when it comes to bias issues in ML models, they are definitely not the only factor. Many times, fairness issues cannot be directly addressed in the data pipeline by “fixing” the dataset via resampling or reweighting the training data. In real-world scenarios involving human data, databases tend to be biased because they reflect existing inequalities deeply rooted in our own societies [1]. Thus, on many occasions, a perfectly balanced dataset cannot be obtained and algorithmic solutions may come in handy. As discussed in a recent article [4], we need to move beyond the idea that “algorithmic bias is a data problem” and start acknowledging that algorithms are not impartial, and some design choices are better than others. In that sense, the choice of specific model architectures, loss functions, and training strategies plays a fundamental role in amplifying or mitigating potential equity issues because they are meant to induce specific behaviour in our systems. If we are able to define fairness metrics, which can then be incorporated into a loss function, we can train our model to optimize it. But measuring whether an AI system makes fair decisions is not a simple task. Formal definitions of algorithmic justice tend to be mutually exclusive, in the sense that not all of them can be satisfied at the same time, and therefore human decisions about which criteria of justice are to be prioritized become crucial.

When these ML systems are deployed in areas such as justice, health, or job hiring, it is easy to imagine the immediate consequences of biased systems, especially when the asymmetries of our own society creep in through the data and design decisions (often unconsciously) taken by those who carry out these developments. Thus, the role of the *people* in the development team is of paramount importance. Data specialists and programmers are the ones who usually not only perform the choice and curation of the databases but also implement and supervise the training process of the models, choose the tasks to be solved and the performance measures, deploy the systems, and monitor them over time. In all these stages, which constitute the life cycle of an ML system, it is people who make the decisions, and many of those decisions can either generate or mitigate algorithmic biases. For this reason, having diverse teams with members who express different points of view, who can audit both the data and the models, before, during and after the development process, constitutes a fundamental component in the construction of more equitable ML systems.

## Note from the Editors

The Research Parasite Award is usually held at the Pacific Symposium on Biocomputing on the Big Island of Hawaii, but in 2020 it was presented at the virtual event via livestream. The establishment of the award was a reaction to an editorial that presented arguments against data sharing, including that it promoted a system where “research parasites” (those who reuse datasets created by “frontline researchers”) would proliferate. As promoters of data sharing GigaScience Press has each year sponsored the Junior Parasite Award for postdoctoral, graduate, or undergraduate trainees and is again proud to support the award with travel grants and prize money. For more, see the Research Parasite Awards website, https://researchparasite.com/.

## Data Availability

Not applicable.

## Abbreviations

AI: artificial intelligence; ML: machine learning.
